# Small extracellular vesicle-associated surface protein biomarkers: emerging roles, opportunities, and challenges in diagnostics

**DOI:** 10.3389/fbioe.2025.1714972

**Published:** 2025-12-01

**Authors:** Thi Thanh Huong Pham, Hiroaki Sakamoto, Tatsuhito Hasegawa, Chisato Sakamoto, Shin-Ichiro Suye, Han-Sheng Chuang

**Affiliations:** 1 Department of Biomedical Engineering, National Cheng Kung University, Tainan, Taiwan; 2 Department of Frontier Fiber Technology and Science, Graduate School of Engineering, University of Fukui, Fukui, Japan; 3 Fundamental Engineering for Knowledge-Based Society, Graduate School of Engineering, University of Fukui, Fukui, Japan; 4 Department of Chemistry and Biology, National Institute of Technology, Fukui College, Fukui, Japan; 5 Division of Engineering, Faculty of Engineering, University of Fukui, Fukui, Japan; 6 Medical Device Innovation Center, National Cheng Kung University, Tainan, Taiwan

**Keywords:** small extracellular vesicle, surface protein biomarker, transmembrane protein, cancer, biosensor, protein profiling, diagnostic tool

## Abstract

Investigation into the use of small extracellular vesicles (sEVs) or the specific subtype exosomes as diagnostic markers has been growing in both research output and market potential, especially in recent years. Despite these ongoing efforts, there is a lack of understanding of the value of sEV surface protein biomarkers beyond just generic tetraspanins as detected analytes in liquid biopsy. While sEV-encapsulated biomolecules, such as nucleic acids or soluble proteins, have been rigorously studied, dependence on sEV lysis would compromise the sensing robustness and diagnostic efficiency. This review article provides a comprehensive overview of sEV transmembrane proteomic signatures and highlights state-of-the-art sensors aiming towards the goal of early diagnosis and clinical monitoring of disease-associated exosomal surface protein markers.

## 1 Introduction to small extracellular vesicles (sEVs) and exosomes

### 1.1 Background

**FIGURE 1:**
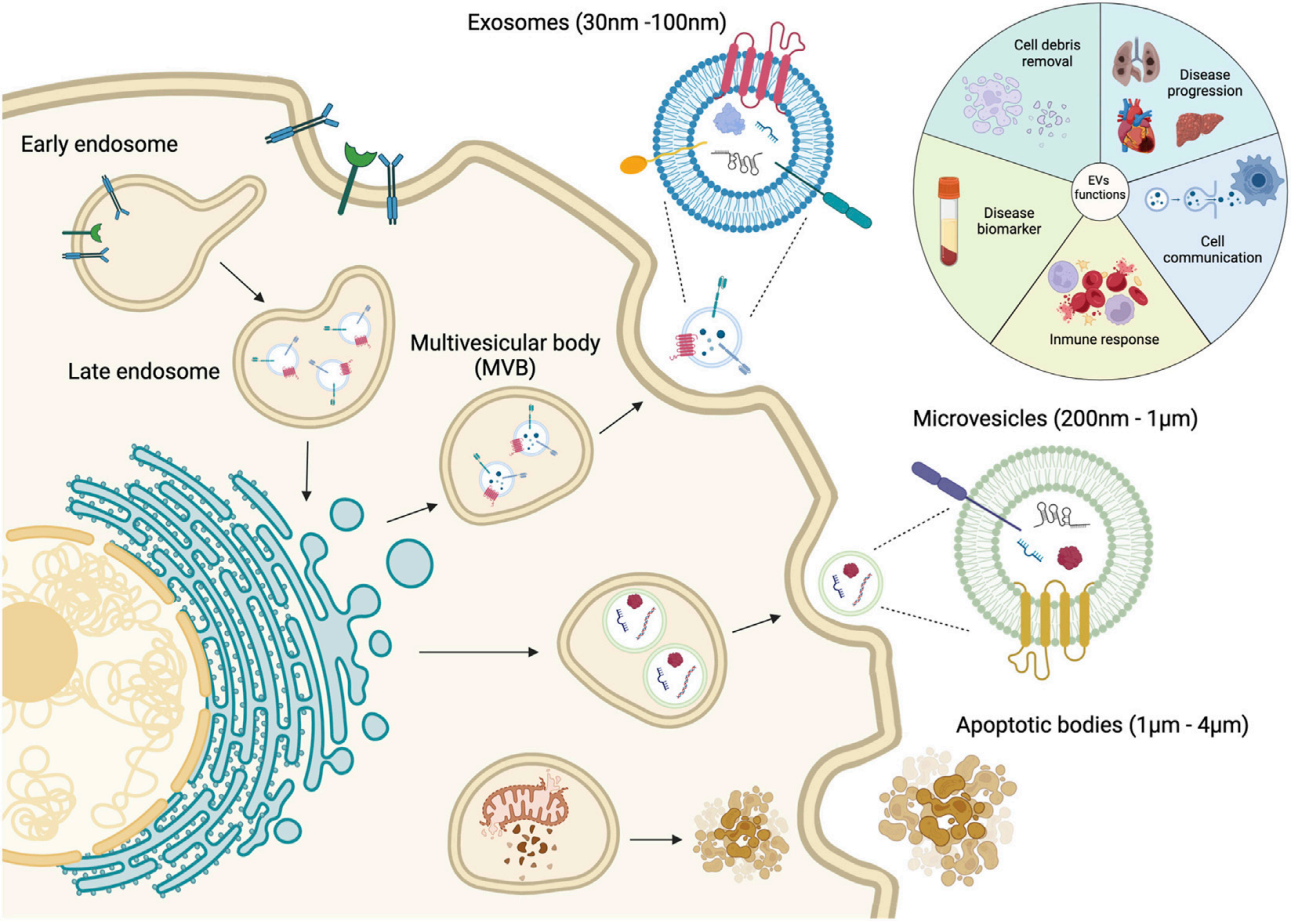
Categories and biogenesis pathways of extracellular vesicles. Figure reproduced with permission (Cano-Carrillo et al., 2024). 2024 MDPI.

Cell-secreted EVs are lipid bilayer-delimited submicron structures categorized into three major subpopulations: apoptotic bodies, microvesicles, and exosomes. This classification is mainly based on biogenesis pathway, size, and specific vesicle biomarkers (Figure 1). The subgroup sEV, which encompasses nanosized vesicles with diameters less than 200 nm, is sometimes used interchangeably with the term ‘exosome’. However, it is important to note that sEVs refer to a more complex population of cell-derived particles, which express their unique biomolecular profiles to separate them from other vesicles and reflect the nature of parental cells. Among these associated molecules, tetraspanin proteins (CD63, CD81, CD9) and Hsp70/90 are considered as exosomal identifying signatures. In addition, this specific subtype of EVs stands out from the rest due to the originating pathway, as they are released from the cell via the fusion of multivesicular bodies or late endosomes with the cellular plasma membrane (Han et al., 2022). In the scope of this review, we respect the terminology used in cited literature, therefore the term, “sEV”, a.k.a. “exosome”, will be used throughout the article for those vesicles that satisfy the following conditions: (1) diameter range between 30 and 200 nm, and (2) expressed exosomal characteristic surface tetraspanins.

Upon release into bodily fluids, these nanoparticles play an important role in various cellular processes. The encapsulated sEV cargoes, including nucleic acids, soluble proteins, or even external particles such as virions, demonstrate diverse biological effects, especially in regulating immune responses (Verma et al., 2023; Wu et al., 2023; Ding et al., 2024; Vahkal et al., 2024; Zhang et al., 2024). These characteristics enable sEVs to serve as highly stable, biocompatible, and minimally immunogenic nanoscale platforms of drug delivery or cell therapy for cancers (Kim et al., 2021; Chen et al., 2023; Li et al., 2024; Wang X. et al., 2024), neurological conditions (Haney et al., 2015; Vahab et al., 2025) and ocular diseases (Verma et al., 2025). Several studies have reported that sEV-derived analytes can serve as diagnostic or predictive markers for disease progression. For instance, sEV-contained miRNAs influence gene expression and subsequent cellular actions via post-transcription mediation, while sEV-associated cytokine release triggers cell proliferation, migration, and cell death. In previous publications, changes in expression of sEV-associated miRNAs have been linked to cancers and other diseases (Sun et al., 2018), cardiovascular disease (Cheng et al., 2018), ischemic stroke (Eyileten et al., 2025), and liver fibrosis (Fagoonee et al., 2025). Elevated sEV-enclosed cytokines were reported in conditions like periodontitis (Liu et al., 2023) and Parkinson’s Disease (Chan et al., 2023). In addition, the role of sEV-encapsulated proteins in cancers, especially breast and lung cancers (An et al., 2019; Lee Y. et al., 2023), has been rigorously studied over the years. These bioactive molecules reflect conditions and characteristics of their parental cells, holding strong diagnostic potential.

**FIGURE 2:**
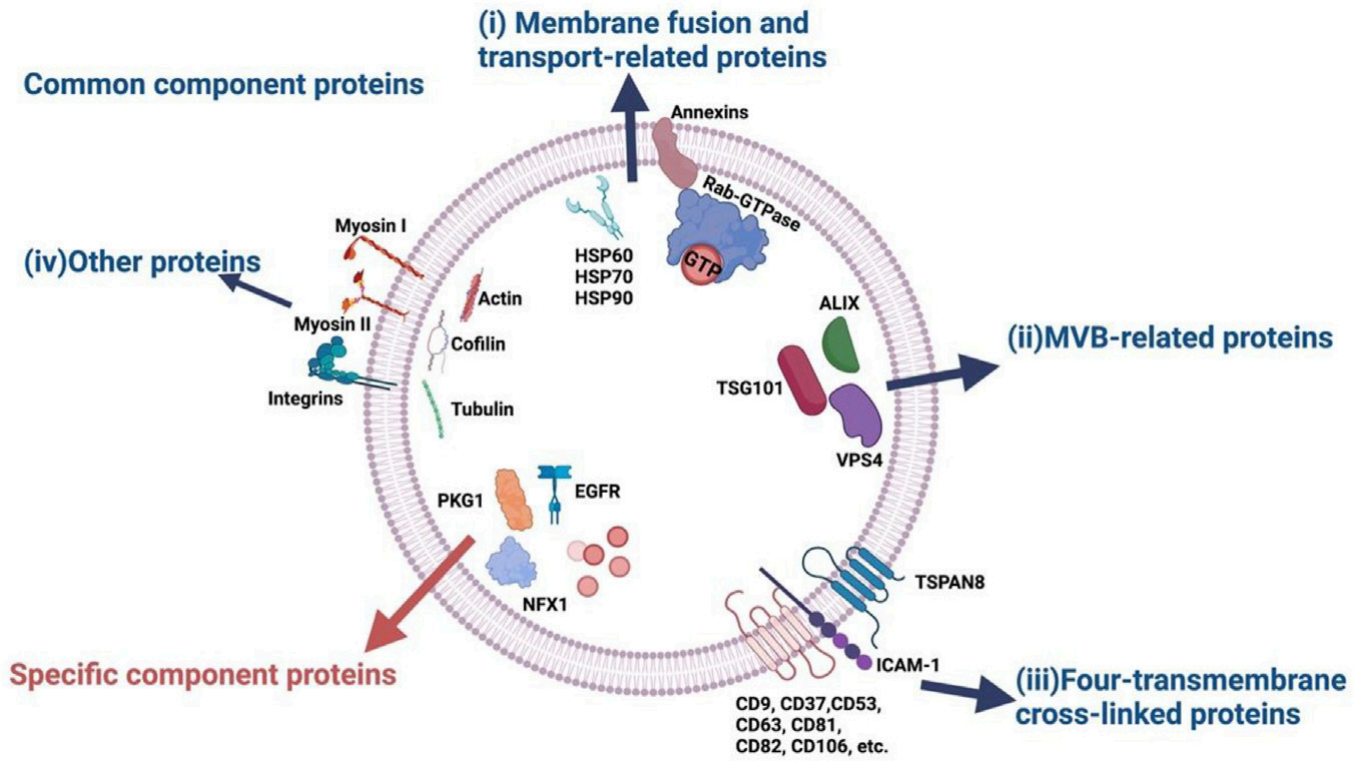
Classes of sEV-associated proteins based on common and specific functions. Notably, sEV membrane-embedded proteins are essential for both roles, including membrane fusion and transport, as well as molecular indicators of parental cells or diseases. Figure reproduced with permission (Li et al., 2023). 2023 MDPI.

On the other hand, the proteins embedded in the vesicular membrane are not for direct uptake but allow interaction between sEVs and their respective recipient cells via receptor binding, and initiate cascades of cellular responses. Structurally, they are associated with the sEV membrane in the same manner as their cellular membrane counterparts, being either peripheral membrane proteins (PMPs) on the outer lipid leaflet or integral membrane proteins (IMPs) that span the entire membrane. These proteins can facilitate common physiological functions of sEVs, such as membrane fusion and transport, or serve as biomarkers of specific cell types or pathological conditions (Li et al., 2023) (Figure 2).

Although sEV internal biomolecules seem to garner more research interests as they directly modulate disease progression, sEV surface markers have the advantage of external exposure, which means experiments can be done without extra sEV lysis. Given their ready availability compared to encapsulated cargoes and unique proteomic profiles, sEV membrane biomarkers are the key to next level diagnostic development. In this review paper, we offer a comprehensive overview of sEV-associated protein biomarkers, highlighting the characterization techniques, and recent advancements in the field of biosensing and diagnostics of these molecules.

### 1.2 sEV isolation methods

**FIGURE 3:**
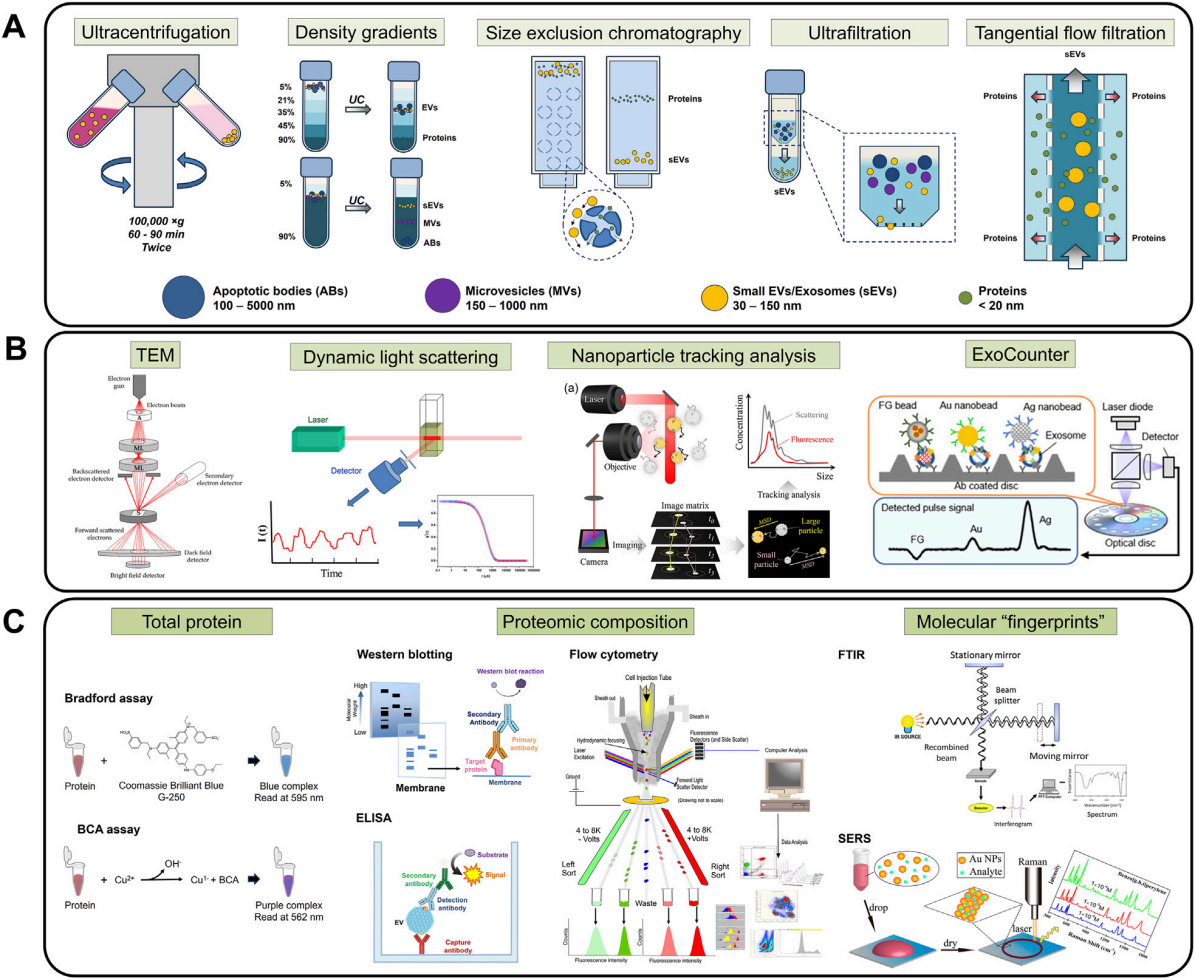
Handling methods for sEV experimentation. **(A)** sEV isolation techniques, namely, UC, DG, SEC, UF, and TFF, are required to ensure sample purity and minimize interfering molecules. **(B)** Physical characterization, such as TEM, DLS, NTA, and ExoCounter, provides an initial evaluation of the physical properties (morphology, size, concentration) of the samples. **(C)** Proteomic characterization protocols like total protein quantification, proteomic composition analysis, and molecular fingerprint scans allow identification and confirmation of biomarkers of interest. Figures reproduced with permission (Xu et al., 2014; Hassan et al., 2015; Malenica et al., 2021; Kwon and Park, 2022; Faramarzi et al., 2023; Ono et al., 2023; Robinson et al., 2023). Copyright 2014 American Chemical Society, 2015 American Chemical Society, 2023 American Chemical Society, 2021 MDPI, 2023 MDPI, 2022 Springer Nature. Art visual elements reproduced from NIAID NIH BIOART Source. Public domain.

Following the Minimal Information for Studies of Extracellular Vesicles (MISEV2023) guidelines, sEV purification and enrichment are typically performed prior to characterization and experimentation (Figure 3A) (Welsh et al., 2024). However, there is no recognized gold standard method for sEV isolation, and the selected technique usually depends on the needs of individual research groups. The quality of sEV isolates heavily relies on the employed method of purification, which can be evaluated based on several properties: size distribution, morphology, and particle concentration. Common sEV extraction approaches include ultracentrifugation (UC), density gradient (DG) ultracentrifugation, size exclusion chromatography (SEC), and ultrafiltration (UF). Accompanying the growth of sEV research, comparison studies of these different techniques on various types of input samples have been extensively conducted in recent years (Askeland et al., 2020; Malvicini et al., 2024; Torres et al., 2024; Hickman et al., 2025). Principles and key differences between these methods are summarized in Table 1.

**TABLE 1:** Isolation methods of sEVs.

sEV isolation technique	Principle	Yield	Co-isolated non-sEVs	Limitations	References
Ultracentrifugation	Applying different centrifugal speeds to collect pellets of various sizes	Low	High	Ultracentrifuge required More prone to sEV deformation Total protein variability	Hickman et al. (2025)
Density gradient ultracentrifugation	Density-based variant of ultracentrifugation	Low	Low	Time consuming Co-isolated lipoproteins and proteins	Yuana et al. (2014)
Size exclusion chromatography	Size-based separation using a porous matrix column	High	Low	Special equipment required Co-isolated lipoproteins	Hickman et al. (2025)
Ultrafiltration	Molecular weight cutoff-based separation using a specialized tube with pores	High	High	Dead-end filtration often results in blocked filter pores after extended uses Co-isolation of free proteins and non-sEVs	Ansari et al. (2024), Romero-Castillo et al. (2024)
Tangential flow filtration	Anti-clogging crossflow filtration technique using hollow fiber membrane and a peristatic pump	High	Low	Special equipment required	Busatto et al. (2018), Visan et al. (2022)
Commercialized isolation kits	Antibody-functionalized bead-based capture and elution	High	Low	Expensive kits Variations in collected samples	Welsh et al. (2024)

UC is a sedimentation-based method that applies different centrifugal speeds to yield corresponding pellets of varied sizes. By modifying centrifugation parameters, several sEV subpopulations can be isolated based on their weight and pelleting rate. While it is widely used among sEV researchers, this approach makes yielded sEVs more prone to deformation and often results in significant total protein variability (Hickman et al., 2025).

DG ultracentrifugation, a density-based variation of high-speed centrifugal isolation, aims to separate EVs into layers using buffers of decreasing density. The nanosized vesicles migrate into fractions due to differences in their buoyant density in prepared gradients, which results in a highly purified isolate.

Similar to UC, SEC also separates EVs based on size using a porous matrix column. Samples are filtered through the matrix-containing column, allowing large-sized particles, i.e., exosomes, to quickly pass through, while smaller protein aggregates are retained. SEC is not only able to purify quality sEVs that express high concentrations of exosomal markers, but the method also shows relatively higher total particle yield and variability. Moreover, cell surface markers were better preserved using this method compared to other approaches (Hickman et al., 2025). However, the above techniques are limited by their time-consuming procedure and low sEV recovery rate.

In addition to these well-established protocols, UF-based methods have also been developed and optimized to improve isolation efficiency. A study evaluating conventional isolation methods (including UC, UF with precipitation, and SEC) from human conjunctival tissue concluded that UF was the most efficient for sEV recovery (Romero-Castillo et al., 2024). In UF, porous membranes are used to entrap nanoparticles of desired size by selecting suitable molecular weight cut-off values. However, this approach may lead to co-isolation of free proteins and non-sEVs, resulting in a large size distribution of the final collection (Ansari et al., 2024). Moreover, dead-end filtration in UF often results in blocked filter pores after extended use.

Alternatively, tangential flow filtration (TFF) is an anti-clogging crossflow filtration technique that utilizes hollow fiber membranes and a peristaltic pump for parallel sample flow relative to the filter pores (Busatto et al., 2018). Compared to UC, TFF demonstrated better performance in terms of yield and consistency (Visan et al., 2022). On the other hand, numerous commercialized kits for EV isolation are accessible, providing a relatively quick and easy way to obtain sEVs of interest from various types of samples, from cell-conditioned medium to human biofluids. Nonetheless, MISEV2023 advised caution and highlighted considerations in using these commercialized products due to risks of impurities and significant variations in collected EV subpopulations.

### 1.3 Characterization techniques

To assess the efficiency of sEV purification and reconcentration, follow-up characterization analysis is essential. Since the properties that define sEV quality span from outer physical traits to vesicular biomarker profiles, there is no single method that can completely characterize isolated sEVs. The following sections introduce conventionally used single modal techniques for sEV assessment and quality testing (Table 2), as well as novel integrated systems.

**TABLE 2:** sEV characterization techniques.

Category	Technique	Principle	Advantages	Disadvantages
Physical characteristics	Electron microscopy (Gardiner et al., 2016)	Using high-energy electron beam to illuminate the sample. Images are formed using the scattered or transmitted electrons	Single sEV identification and morphology check Standardized structures observed (cup-shaped or spherical depending on imaging modes)	Non-quantitative Time consuming Preprocessing required
	Dynamic light scattering (Lawrie et al., 2009)	Brownian motion-based method in which monochromatic light is projected onto samples, causing light scattering and intensity fluctuations	Average particle size and distribution are derivable with known viscosity and temperature Minimal preprocessing Time saving	Non-quantitative Low resolution
	Nanoparticle tracking analysis (Dragovic et al., 2011; Comfort et al., 2021)	Brownian motion tracking through light scattering after illuminating onto samples from a laser source	Particle concentration and size distribution are derivable with better resolution Minimal preprocessing Time saving Fluorescent detection can be implemented	Unable to separate sEVs and non-sEVs High variations during operation
	ExoCounter (Kabe et al., 2018; Ono et al., 2023)	Combining particle counting and immunolabelling using antibody-modified capture wells and antibody-modified nanobeads	Particle concentration and size are measurable High specificity and accuracy	Low throughput
Total molecular properties	Total protein analysis (Bradford, BCA) (Olson, 2016)	Detection of proteins via colorimetric assays	Rapid and easy procedure Cheap reagents	Overestimation of concentration No profiling power Unable to separate sEV-associated molecules and non-sEV analytes
	Lipid composition (separation analysis) (Skotland et al., 2019)	Separation of lipid for mass-spectroscopy analysis based on polarity (thin-layer chromatography) or volatility (gas-liquid chromatography)		
	Nucleic acid analysis (capillary electrophoresis) (Zeringer et al., 2013)	Size and charge-based separation of nucleic acids using capillary tubes and electric field		
	Molecular fingerprint analysis (FTIR, SERS) (Shin et al., 2018; Chalapathi et al., 2020; Shin et al., 2020; Wong et al., 2022)	In FTIR, sEV molecular contents absorb infrared light at characteristic wavelengths corresponding to specific molecular bond vibrations, while SERS is based on Raman peaks and intensities due to scattered laser light upon illumination onto samples on metal	Label-free, lysis-free procedure Provides sEV chemical conformation and composition analysis	

#### 1.3.1 Physical characteristics

Particle morphology, number and size are among the basic parameters verified by sEV characterization (Figure 3B). Given their nanoscopic size, electron microscopy (EM) is essential to visualize sEV phenotypes. Several variations of EM, especially transmission EM (TEM), have been consistently employed in EV studies as this technique allows individual vesicle identification and size determination (Gardiner et al., 2016). For instance, it is well-known that sEVs appear as cup-shaped structures in negative staining TEM image (Thery et al., 2006; Jung and Mun, 2018), while in cryo-TEM, spherical structures are observed (Raposo and Stoorvogel, 2013), due to the differences in imaging conditions.

Dynamic light scattering (DLS) is a Brownian motion-based method that can determine the size of biological vesicles in aqueous buffers. DLS projects monochromatic light onto samples containing particles, which in turn scatter the light and cause intensity fluctuations. With known viscosity and temperature, average particle size and distribution can be estimated via Stokes-Einstein equation (Lawrie et al., 2009). However, it is impossible to derive sEV quantity from EM or DLS.

On the other hand, nanoparticle tracking analysis (NTA) is a newer, high-throughput method that is widely used for visualization and quantification of EV populations. Based on the phenomenon of scattering light from a laser source when directed onto EVs, the random thermal-induced Brownian movements of the nanosized vesicles are tracked and thus, particle concentration and size distribution of the sample population are calculated (Dragovic et al., 2011; Comfort et al., 2021). In recent years, fluorescence detection has been implemented in NTA instruments, enhancing accuracy and allowing in-depth analysis of true sEV fractions (Desgeorges et al., 2020; Dlugolecka et al., 2021).

While the above techniques are applicable to all submicron particles of all materials, a unique platform for the sole purpose of quantifying biological vesicles, ExoCounter, was developed by combining particle counting and immunolabelling. The device consists of antibody-modified capture wells and antibody-modified nanobeads, allowing sEV capture in a sandwich format and detection via optical pick up (Kabe et al., 2018; Ono et al., 2023).

#### 1.3.2 Molecular properties

EVs consist of various subgroups originating from different cell sources via distinctive biogenesis pathways, which directly influence their molecular expression. For exosomes, the endosomal machinery distributes cell-produced biomolecules to each individual vesicle, encompassing membrane and encapsulated contents. Thereby, conventional methods frequently used to assess these molecular entities can also be applied to sEV-associated counterparts, often with certain adaptations.

Depending on the experimental target, the total levels and profiles of protein, lipids, and nucleic acids (e.g., miRNA) are examined using molecule-specific methods. For example, total protein data is often acquired by well-established protocols such as Bradford and BCA assays (Olson, 2016) (Figure 3C); lipid composition is usually studied using separation analysis, such as thin-layer chromatography, gas-liquid chromatography, and mass spectrometry (MS) (Skotland et al., 2019); and sEV nucleic acids are recovered via capillary electrophoresis (Zeringer et al., 2013). However, total composition analysis often results in concentration overestimation due to the heterogeneity of sEV isolates, especially in cases where co-isolated non-sEVs are present (Welsh et al., 2024). Moreover, the final readout greatly depends on sample preparation and the chosen assay for analysis, leading to questions on reproducibility among sEV researchers (Vergauwen et al., 2017).

Since sEVs have prognostic and diagnostic values because of their molecular composition, primarily proteins and RNAs, it is crucial to understand the individual contributions of these biomarkers. Hence, for an in-depth investigation, further downstream analysis is required to detect, quantify and/or amplify a specific target of interest. Conventionally, enzyme-linked immunosorbent assay (ELISA), Western blot (WB), and flow cytometry (FC) are commonly used in sEV protein profiling studies (Properzi et al., 2013) (Figure 3C), while polymerase chain reaction (PCR) variants and high-throughput sequencing protocols are useful tools to assess sEV-derived nucleic acids (Moldovan et al., 2013; Kumar et al., 2020).

Although not molecule-specific, Fourier-Transform Infrared (FTIR) spectroscopy and Surface-enhanced Raman Scattering (SERS) offer a label-free, lysis-free chemical conformation and composition analysis of sEVs. The vesicle-associated molecular contents are revealed through specific “fingerprints”, i.e., absorption spectrum and related derivations such as ratio mixtures of different bands for FTIR, or Raman peaks and intensities in SERS (Shin et al., 2018; Chalapathi et al., 2020; Shin et al., 2020; Wong et al., 2022) (Figure 3C).

#### 1.3.3 Multimodal systems

On the other hand, multimodal approaches provide a comprehensive understanding of sEV proteomics. In these setups, standalone techniques for distinct sEV parameters are integrated into a single workflow, allowing direct isolation and multiple downstream assessments of the same sample in one unified physical system.

EV-Ident is a nanoporous multi-membranal microfluidic device coupled with *in situ* fluorescent labeling which performs EV size-based enrichment and surface protein analysis simultaneously. By separating EVs into respective size fractions, subpopulations of interest can be further investigated for detailed physical and biochemical features (Kim et al., 2020). Another notable example is the multiparameter analysis of various EV properties including surface marker profiling in single apparatus designed by Normak et al. (Normak et al., 2023). The system incorporated liquid chromatography (LC) for EV separation, multi-angle light scattering for size and concentration determination via angular dependence and light scattering intensity, and fluorescent detection for total protein quantification from tryptophan residue intrinsic signal and identification of membrane-bound CD81 through Alexa Fluor 488-labelled antibodies. By combining single methods together using microfluidic approach, these novel platforms achieve enhanced analytical power using less sample volume and optimizable for high throughput.

### 1.4 sEV-associated surface proteins as potential diagnostic targets

The role of EVs has been progressively clarified towards a more thorough understanding of this analyte through numerous publications in recent years. Many review articles have discussed the advantages of sEVs and exosomes as a powerful cancer diagnosis and treatment tool (Kalluri and LeBleu, 2020; Chen et al., 2024; Ma et al., 2024). Despite the rarely successful translation into approved clinical applications, their potential in diagnostics, prognostics, preventive medicine, and treatment is undeniable.

It is well-known that EVs and sEVs play an important role in cell-cell interactions, signal transport, and mediating pathophysiological pathways, such as inflammation (Buzas et al., 2014; Useckaite et al., 2020), cell death (Sanwlani and Gangoda, 2021; Yang et al., 2024), metastasis (Patel et al., 2025), and more. In spite of the protective effects of EVs during immunoregulation (Wei et al., 2024), abnormal elevation in sEV production rate is a prominent sign of disease progression, as reported in several papers investigating cancers (Zhang et al., 2015; Konig et al., 2017; Bebelman et al., 2021), osteoporosis (Zhang et al., 2024), epilepsy with depression (Yakovlev et al., 2023), periodontitis (Liu et al., 2023), preeclampsia (PE) (Bowman-Gibson et al., 2024), and more. In contrast, there are cases where sEV level drop associates with worsening conditions, such as the role of circulating endothelial cell-derived EVs in systemic sclerosis (SSc) and endothelial damage (Argentino et al., 2024).

Although tracking the total sEV abundance reveals the intricacies of tumorigenesis and tumor microenvironments that conventional clinical routines fail to identify, a solely enhanced sEV concentration often appears systemic and less disease-specific (Xu et al., 2024), thereby dampening the potential for diagnostic applications. While sEV cargo could be a more suitable alternative, since protein and nucleic acids contents in each sEV closely resemble the parental cells, assessment of these encapsulated biomolecules requires the lysis of the vesicle membrane, adding to the tedious workload.

Switching to the abundant, disease-reflective sEV-associated surface biomarkers would be a promising approach to overcome the above challenges. These proteins are accessible from the outer surface of the vesicles, which reduces the need for preprocessing or lysis to minimum, thereby saving both time and reagents. As a result, the structure integrity of sEVs can be well preserved during and after analysis, allowing recovery of intact sEV for further tests. Moreover, the localization advantage of sEV-associated surface proteins enables direct detection, making them highly suitable for point-of-care (POC) diagnostic approaches.

Families of sEV-specific transmembrane proteins, i.e., tetraspanins, are intensively investigated in sEV population characterization or cell line phenotyping studies (Breitwieser et al., 2022; Giovanazzi et al., 2023; Rydland et al., 2023). However, they are not as meaningful in the diagnostic aspect, which requires further investigation into the identification of unique disease-associated sEV surface biomarkers. For example, classic molecular indicators of cancer, such as EpCAM and PD-L1, can be expressed on sEV surface to serve their immunosuppressive and tumor progressive functions (Hu et al., 2023; Sfragano et al., 2023). Such sEV-surface associated biomarkers are the key factors towards advancement in diagnostic tool development.

## 2 Characterization and profiling of sEV-associated surface proteins

### 2.1 Extraction of sEV-associated surface proteins

Generally, in cell membrane protein purification, target proteins are released from the membrane and solubilized in suitable buffers (Wu and Yates, 2003). Due to structural and localization differences of extrinsic PMPs and intrinsic IMPs, specific procedures of membrane protein collection must be carried out for downstream proteomic analysis. For example, PMPs are loosely linked to the lipid bilayer via non-covalent bonds such as electrostatic forces, hydrogen bonds, or hydrophobic interactions. As a result, extraction of PMPs can be achieved using highly concentrated salt solutions or alkaline/acidic buffers to disrupt these weak linkages. On the other hand, IMPs, with a higher degree of association with the membrane, require membrane rupture for release by either ionic, non-ionic, or zwitterionic detergents. The selection of detergents can affect the efficiency of subsequent processes as well as the integrity of the proteins of interest. Therefore, it is highly recommended to review experimental factors and employ detergent removal techniques (Smith, 2017).

Notably, sEV membranes share a high structural and functional resemblance to cell membranes, including the lipid bilayer structure with embedded proteins and other molecules. While the above framework is transferable to sEV membrane proteomics, several challenges still persist, namely, the relatively low concentration, aggregation tendency, and vulnerability of membrane proteins to proteases. Xu et al. reviewed some exosome-focused membrane protein enrichment techniques, including sodium carbonate fractionation/Triton X-114 phase partitioning and mild proteinase K digestion (Xu et al., 2019). Through sodium carbonate fractionation/Triton X-114 phase partitioning, isolation of PMPs and IMPs is favored while cytosolic and organellar soluble proteins are depleted (Kim et al., 2015). Furthermore, proteolytic treatment with proteinase K, which is relatively less specific than trypsinization, identifies a wider range of surface-exposed IMPs, revealing potentially meaningful epitopes as disease biomarkers (Skliar et al., 2018).

### 2.2 Conventional methods

A vast number of diseases, including cancers, develop in a multi-staged manner involving various biomarker fluctuations during pathological staging and progression (Matejova et al., 2023). These changes are reflected in the expression on secreted sEVs, further complicating the already complex sEV proteomics. Hence, it is crucial to carry out intensive profiling studies of sEVs to effectively identify potential biomarkers for diagnosis and prognosis (Supplementary Figure 1). In recent years, this task has been tackled by numerous research groups with expanded focus on different diseases, adapting well-established methods for cell-free proteins to sEV-associated proteins (Table 3).

**TABLE 3:** Conventional and emerging sEV-associated protein characterization and profiling techniques.

Category	Technique	Key application	sEV lysis required	High throughput	Multiplexing capacity	Major challenges
Conventional methods	WB (Coumans et al., 2017)	Identification of targeted protein biomarkers	Yes	No	No	Weak quantitative power Requires high concentration
	ELISA (Coumans et al., 2017)	Quantitative detection of targeted protein biomarkers	Optional	Yes	No	Requires high concentration
	MBFCM (Li et al., 2022; Nguyen et al., 2024)	Protein profiling based on a preselected biomarker panel	No	Yes	Yes 37 markers	Complex data interpretation
	LC-MS/MS (Shao et al., 2018; Bandu et al., 2019)	Quantitative profiling for biomarker discovery	Yes	Yes	Yes	Interference due to non-sEVs
Emerging methods	Oligonucleotide tags (Wu et al., 2019; Shibata et al., 2024; Steiner et al., 2025)	Short nucleotide sequences serve as detection probe or both capture and detection probes	No	Yes	Yes 38 proteins	Requires additional generating and selective processes for highly specific oligonucleotide sequences
	SMLM (Martorana et al., 2024)	Superior resolution imaging-based profiling using fluorescent dyes	No	Yes	Yes Co-localization	Photobleaching Phototoxicity
	DISVT (Amrhein et al., 2023; Taylor et al., 2024)	Dual fluorescent tagging in combination with conjugated gold nanoparticles as detection probe for dark field imaging-based detection	No	Yes	Yes	Requires imaging equipment and ML pipelines for analysis

#### 2.2.1 Traditional bulk immunoassays: WB and ELISA

WB and ELISA are among the most widely used methods in sEV proteome characterization due to significant advantages, such as high availability, acceptable costs, and high suitability for sEV studies. Until now, these techniques still retain their relevance and effectiveness in investigations of biochemical pathways and studies of pathological marker identification with clear and established frameworks (Kowal et al., 2017).

WB has a low dynamic range of roughly 100:1, hence having weak quantitative power. In WB, the protein content is confirmed via a multistep process, including sEV lysis, gel electrophoresis, and immunolabelling after membrane transfer. For instance, to identify valuable sEV surface markers for breast cancer, Huttmann et al. employed WB for extended investigation following a co-localized protein expression study (Huttmann et al., 2024). This approach finalized ST14 and CLDN3 as potential sEV-based breast cancer markers. In a study by Kim et al., the surface glycoprotein CD109 was reported to be an ovarian cancer biomarker as its concentration elevated in both soluble and sEV-associated forms, validated in ovarian cancer stem cell spheroids and patient samples using WB (Kim et al., 2024).

The widely commercialized ELISA performs on the principle of antigen-antibody binding, providing a highly specific detection in a high throughput assay format. Typically, sEVs are captured via immobilized antibodies on ELISA microwell surface and detected by secondary antibodies coupled with an effective signal amplification. Aparicio et al. confirmed sEV-associated PF4 and C1R as plasma biomarkers of sarcopenia using ELISA post extensive proteomic analysis (Aparicio et al., 2024). In addition, ELISA-inspired protocols for detection of EVs are developed and published in recent years, showing the method’s continued adaptability in modern biomedical research (Lee et al., 2019; Takizawa et al., 2022; Driedonks et al., 2024).

Despite the obvious strengths, there are various drawbacks that researchers should consider before conducting these bulk immunoassays. For instance, WB and ELISA can suffer from reduced sensitivity when analyzing vesicle-bound proteins instead of free-flowing molecules. In addition, they are time-consuming, not designed for multiplexing, requiring large volume as well as highly concentrated samples for sufficient signal readout (Coumans et al., 2017).

#### 2.2.2 Bead-based multiplexed sEV analysis by flow cytometry

FC is a well-established method for cellular analysis, allowing for identification and quantification of single cells and submicron particles. However, the major challenge in analyzing sEVs using FC is due to their nanoscale size. On a limited surface area combined with a highly heterogeneous expression of surface epitopes, conventional fluorescent labelling is often insufficient for a definite readout, not to mention significant background noise (Gul et al., 2022). In an attempt to characterize plasma-derived EVs from glioblastoma (GBM) patients and normal donors across a panel of biomarkers using spectral FC, Aibaidula et al. developed a staining protocol specialized for EV membrane (Aibaidula et al., 2023). Their results highlighted the differences between GBM-derived EV and non-neoplastic EV populations, in which GBM patients showed highly heterogeneous tetraspanin expression and increased CD11b.

To enhance the power of this technique in nanosized sEV characterization, a bead-based approach is coupled with conventional FC (Balbi et al., 2019; Yang et al., 2020). In general, EV-specific antibody-modified polystyrene beads are employed for EV capture, followed by staining with color indicator-bound secondary antibodies. Subsequent laser-based illumination allows for fluorescent detection and flow analysis. Commercialized multiplex bead-based EV flow cytometry (MBFCM), marketed as MACSplex, has been discussed, optimized, and widely used in numerous EV studies. Generally, samples containing EVs are incubated with target antibody-conjugated beads for capture and then detected by fluorescent detection antibodies against a panel of 37 biomarkers, including tetraspanins (CD63, CD81, etc.). Publications reporting MACSplex results of disease-related EV surface markers are summarized in Table 4.

**TABLE 4:** sEV protein profiling studies using MBFCM (MACSplex).

Disease/condition(s)	Sample type(s)	Identified potential biomarkers
Antiphospholipid syndrome (Stok et al., 2020)	Plasma sEV	CD8, CD44, CD62P, CD133/1
Breast cancer (Ekstrom et al., 2022)	Lymphatic drain fluid sEV	CD24, CD29, CD44, CD146
GBM, Multiple sclerosis (MS) (Brahmer et al., 2023)	Cell line-derived sEV Plasma sEV	CD44, CD90, ^a^CD36, CD54, CSPG4, GD2, CD13, CD49e, CD49f (GBM) ^a^GALC, CD68, CD29, CD107a, CD49a (MS)
GBM (Franceschi et al., 2024)	Tumor explant-derived sEV	CD105, CD133/1, CD14, CD142, CD146, CD29, CD44, CD56, HLA-DR/DP/DQ, MCSP
GBM (Serban et al., 2024)	Plasma sEV	CD33, CD133, EpCAM, SSEA4
Hemorrhagic shock (Weber et al., 2023b)	Plasma sEV	CD44, CD33
HIV/HCV coinfection (Martinez-Gonzalez et al., 2020)	Plasma sEV	HLA-DR/DP/DQ, CD81, CD8
Idiopathic pulmonary fibrosis (IPF) (d'Alessandro et al., 2021)	Serum sEV	CD19, CD69, CD8, CD86, CD209, CD133/1, MCSP, ROR1, CD42a
Interstitial lung diseases (ILD) (d'Alessandro et al., 2023): IPF, Hypersensitivity pneumonitis (HP), Sarcoidosis	Bronchoalveolar lavage sEV	CD56, CD105, CD142, CD31, CD49e (IPF) CD86, CD24 (HP) CD11c, CD1c, CD209, CD4, CD40, CD44, CD8 (HP/sarcoidosis) CD19, CD45 (IPF/sarcoidosis)
Laryngeal cancer (Bocchetti et al., 2024)	Serum sEV	CD1c, CD2, CD3, CD4, CD11c, CD14, CD20, CD44, CD56, CD105, CD146, CD209
Osteoarthritis (Matejova et al., 2023)	Plasma sEV	CD45, CD326, CD56
Ovarian cancer (Pantazi et al., 2025)	Cell line-derived sEV	CSPG4, CD105, CD146, ^a^SSEA-4, ^a^CD142, ^a^EpCAM
Pediatric idiopathic nephrotic syndrome (Cricri et al., 2024)	Serum sEV Urine sEV	CD41b, CD105, CD29 (Urine); CD146+ serum EV and CD29^+^ urine EV
Polytrauma injury (Weber et al., 2023a): Predominant abdominal, chest, brain injuries	Plasma sEV	CD42a (Polytrauma) CD209 (Abdominal) CD11 (Chest) CD62P (Brain)
Prostate cancer, benign prostatic hyperplasia (Salvi et al., 2021)	Serum sEV Urine sEV	CD9, CD63, CD24 (Urine) CD62P, CD41b, CD29, CD42a, CD31 (Serum)
Systemic lupus erythematosus (Chuang et al., 2022)	Serum sEV	CD9, CD63, CD62P, CD45
SSc with complications (Tonello et al., 2025): Pulmonary arterial hypertension (PAH), ILD	Serum sEV	CD146, ^a^CD42a, CD29, HLA-ABC (SSc); ^a^CD3, CD56 (SSc/PAH)
Traumatic spinal cord injury (Hörauf et al., 2024)	Plasma sEV	^a^CD47, CD56, CD68, ADAM17

^a^ Most significant biomarker(s).

Based on the principles of MACSPlex kit, Nguyen et al. proposed a modified version of MBFCM specialized for protein profile assessment of mesenchymal stromal cell (MSC)-derived EVs, with the major change being the two MSC biomarker panels (Nguyen et al., 2024). This work successfully identified a set of either positively or negatively presented surface protein markers on MSC-derived EVs, which showed potential to distinguish between EVs isolated from different sources. Aside from that, another commercialized immunoassay based on FC principle, LuminEV, was designed to profile sEV-specific tetraspanins (CD9, CD63, and CD81). This assay can detect other cell line-associated EV markers, namely, CD235a, GAP43, and CD68, for multiplexed specificity assessment and colocalization study. The kit-included MagPlex colored microspheres are covalently modified with antibody to bind with target EVs, followed by the detection by a cocktail of biotinylated antibodies.

MBFCM follows a sandwich conformation, which incorporates the binding of EVs on capturing beads and secondary antibodies, enables a highly specific sensing performance with minimal non-EV triggering signals. This advantage allows for reduction of pre-processing human biofluids, hence facilitates the procedure time (Li et al., 2022). Despite the impressive performance, MBFCM results can be tricky to interpret, as a “not clearly positive” marker does not necessarily mean that it is totally absent from the EV surface. Due to the nature of EVs, a negative readout can result from low abundance of EV-bound proteins or insufficient quantity of EVs, which requires extended investigation to confirm (Nguyen et al., 2024).

#### 2.2.3 Liquid chromatography tandem mass spectrometry

MS-based methods, especially liquid chromatography tandem mass spectrometry (LC-MS/MS), have been extensively used in sEV protein profiling as described in previous reviews (Shao et al., 2018; Bandu et al., 2019). Following sEV lysis and protein digestion, peptides are separated via LC and ionized by electrospray prior to MS analysis, in which the processed peptides are passed through mass analyzers, thus separated based on mass-to-charge ratio (Guo et al., 2025). A bottom-up proteomic profiling using LC-MS/MS analysis evaluated isolated EVs from T cells and natural killer cells of large granular lymphocyte leukemia (LGLL), revealing the dynamics under LGLL cytotoxicity (Ploeger et al., 2024). Similarly, Han et al. analyzed the protein composition of lung-derived EVs by LC-MS/MS to investigate the marker variations due to different methods of tissue treatments and dissociation (Han et al., 2024). Cvjetkovic et al. completed a global quantitative proteomic analysis through LC-MS/MS to screen for colon cancer biomarker located on tissue EV surface (Cvjetkovic et al., 2024). Among over 2000 proteins analyzed, 53 proteins were identified as over-expressed in colon cancer tumor-derived EVs.

Several technical advancements have been established in the past years to improve the efficiency of conventional LC-MS/MS for sEV-applicable proteomics studies. For instance, ultra high-pressure LC outperforms conventional LC in terms of resolution, sensitivity, separation time, and number of profiled proteins, which is greatly beneficial for low protein-abundance analytes like sEVs. Conventional MS does not allow single nanovesicle analysis due to low sensitivity; however, an advanced technique like nanoprojectile secondary ion MS can enable multiplexed individual EV characterization (Lee S. et al., 2023). On the other hand, one major drawback of LC-MS/MS is the bias of sample purity. This critical property of sEV isolates is hampered due to the complex nature of sEV populations in human bodily fluids, and concurrently, the wide selection of sEV isolation methods without a defined gold standard adds to the challenge. As a result, it is recommended for careful considerations prior to carrying out experiments (Pocsfalvi et al., 2016).

### 2.3 Emerging profiling techniques

**FIGURE 4:**
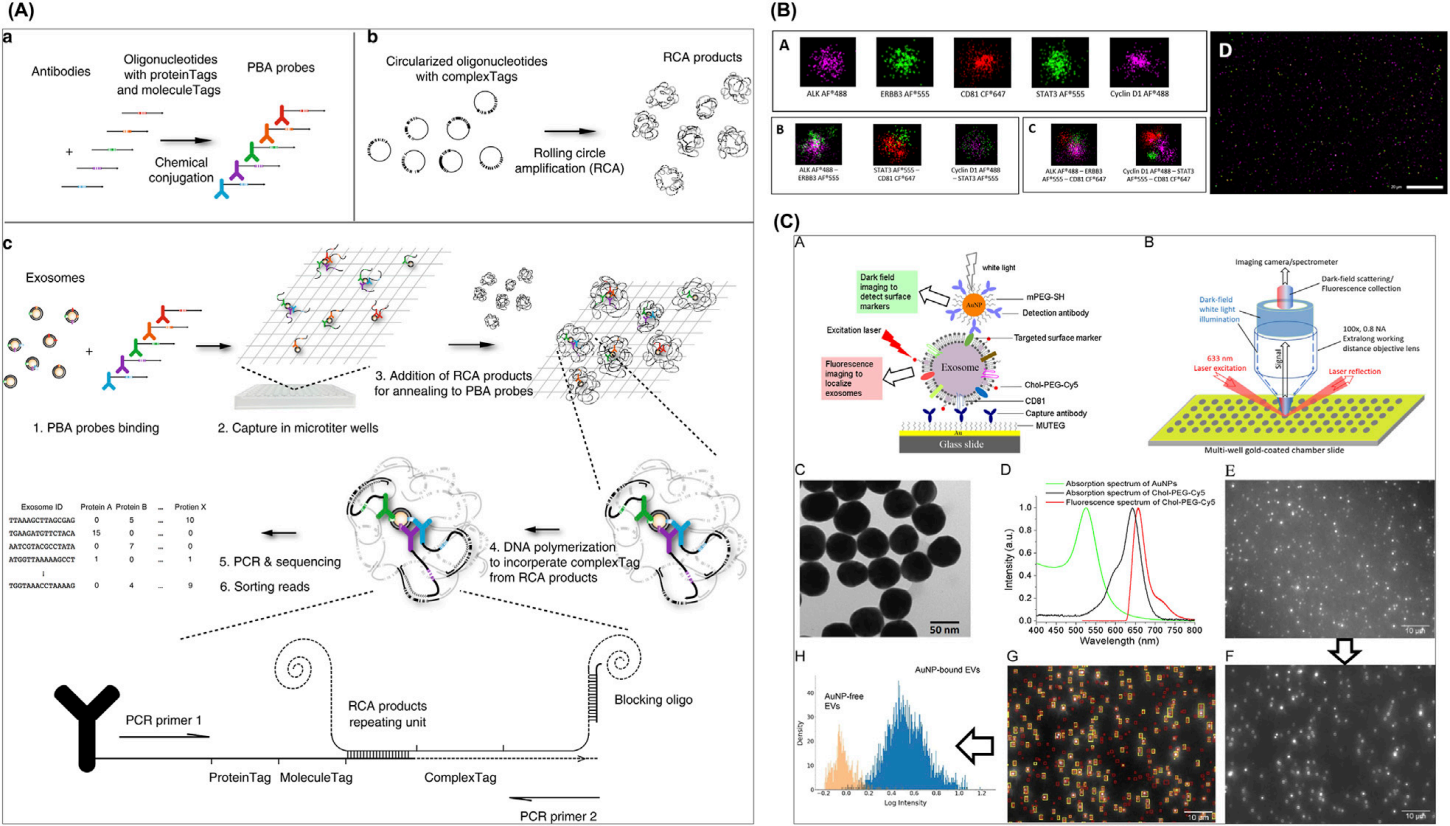
Emerging sEV-associated protein profiling approaches. **(A)** A PBA for sEV surface protein identification. **(B)** dSTORM with ONI-manufactured EV profiler kit was employed to confirm EV-specific surface markers CD81 with the co-expression of several blood-derived prostate cancer transmembrane biomarkers ERBB3 and ALK. **(C)** DISVT approach in which EV membrane is decorated with laser excitation-detected fluorescent tags for a report of the total EV number, while selected antibody (anti-HER2, anti-EpCAM, or anti-CD24)-conjugated AuNPs are employed as a detection probe for breast cancer-associated EVs. Figures reproduced with permission (Wu et al., 2019; Amrhein et al., 2023; Martorana et al., 2024). Copyright 2023 American Chemical Society, 2024 MDPI, 2019 Springer Nature.

Traditional bulk immunoassays, MBFCM, and LC-MS/MS have been conventionally used since the dawn of sEV research, demonstrating their continued versatility, essential role, and significance in the field. However, aiming for higher efficiency, easier handling protocols, and high-throughput system, several alternative sEV-associated protein profiling methods have been developed (Figure 4; Table 3).

#### 2.3.1 Oligonucleotide tags

In DNA-assisted immunoassays, short nucleotide sequences, or DNA barcodes, serve as identifiers for molecules of interest (Nong et al., 2012). By conjugation with selective antibodies, these amplifiable tags can provide highly sensitive, specific, and multiplexed sensing of proteins, showing enormous potential in sEV surface marker profiling.

In an effort to avoid the limitations of conventional FC in EV studies, Shibata et al. proposed a “tag method” for detection and quantification of EV surface proteins (Shibata et al., 2024). Using antibody-conjugated magnetic beads as capture probe and antibody-specific oligonucleotides as detection probe, following EV binding, oligonucleotide tags were collected and assessed by quantitative PCR (qPCR). Their work successfully characterized expression profile of the cancer hallmark marker CA-9-19 and tissue factors on pancreatic cancer cell-derived EVs.

Wu et al. reported a proximity-dependent barcoding assay (PBA) for sEV surface protein identification (Wu et al., 2019). In this approach, 8-nucleotide proteinTag and 8-nucleotide moleculeTag were attached to antibodies to form PBA probes; and another complexTag, composed of a 15-nucleotide circular DNA, was subjected to rolling circle amplification (RCA) to create a library of RCA products. Following incubation of sEVs with PBA probes, prepared RCA products hybridized with sEV-bound PBA probes, generating the only proximity barcode that can be amplified for sequencing later. Lastly, tag sorting reads reveal the protein composition on assessed individual sEVs. This multiplexed assay achieved a successful profiling for a 38-protein panel to identify unique protein combinations representing different sEV populations.

On the other hand, a work by Steiner et al. aiming to establish urinary EV protein profiles utilized proximity extension assay (PEA), which not only revealed the protein signature of urinary EVs, including upregulation of potential marker proteins such as MMP12, MMP7, and HO-1, but also indicated its association with bladder cancer staging (Steiner et al., 2025). In the Olink-commercialized technique PEA, two separate DNA-oligonucleotide-tagged antibodies bind to the protein target, leading to close proximity DNA hybridization, i.e., the formation of a DNA barcode, which is finally amplified by PCR and analyzed by next-generation sequencing or qPCR.

#### 2.3.2 Single molecule localization microscopy

Another innovative approach is Single Molecule Localization Imaging Technology (SMLM), a category of Super-Resolution Imaging Technology. Although widely used for cell and sEV imaging and characterization, they are lesser known for sEV profiling. In recent years, several articles have reported the potential of direct stochastic optical reconstruction microscopy (dSTORM), a SMLM-based method, in sEV proteomics studies. This technique provides a superior resolution of below 50 nm to reconstruct high-quality images via localization of photoswitching from a single fluorophore (Rust et al., 2006), thereby outperforming conventional fluorescent microscopy (Ghanam et al., 2023). As a result, dSTORM effectively visualizes single protein profiles or co-localized markers in EV populations through multicolor fluorescent dyes. However, photobleaching or phototoxicity impedes the method by reducing the image capturing period.

In a work by Martorana et al., dSTORM with ONI-manufactured EV profiler kit was employed to confirm EV-specific surface markers CD81 with the co-expression of several blood-derived bioindicators of prostate cancer, including transmembrane ERBB3 and ALK, as well as intra-vesicular STAT3 and Cyclin D1 (Martorana et al., 2024). The results indicate that this marker combination is useful to differentiate between prostate cancer and hyperplasia, demonstrating potential as a liquid biopsy test. This method was also applied in a study by Tassinari et al. along with MACSPlex assay for investigation of surface proteins expressed on EVs isolated from extracellular matrix (ECM) of colorectal cancer (CRC) tissue (Tassinari et al., 2024). Their findings revealed varied ECM-EV protein compositions in different cancer stages, with enriched CD24, CD326, CD42a, and CD25 as significant markers of Stage IV CRC, in contrast to low levels in healthy mucosa.

#### 2.3.3 Dual imaging single vesicle technology

Amrhein et al. developed a novel method called Dual Imaging Single Vesicle Technology (DISVT), integrating label-based, dual fluorescent, and darkfield imaging (Amrhein et al., 2023), which was subsequently successfully integrated into an automated, machine learning-powered diagnostic platform by Taylor et al. (2024). In this individual vesicle approach, the EV membrane is decorated with laser excitation-detected fluorescent tags for a report of the total EV number, while selected antibody (anti-HER2, anti-EpCAM, or anti-CD24)-conjugated gold nanoparticles (AuNPs) are employed as a detection probe for breast cancer-associated EVs. By capturing darkfield images and performing analysis using in-house machine learning pipeline, a successful diagnosis and differentiation of HER2-positive breast cancer at Stages II, III, and IV was established based on CD24 and EpCAM expression levels.

## 3 Emerging sensing techniques targeting EV-associated surface proteins for biomedical applications

Profiling techniques reveal the molecular characteristics of EV populations of various sources, thus contributing to the identification of novel disease-associated biomarkers. However, they are not quantitative. Biosensors are devices or assays that convert biological and biochemical signals into readable results indicating the presence and concentration of analytes of interest. Along with the growth of our understanding of sEVs and their association with diseases, the development of sEV sensors is rapidly advancing. In this section, state-of-the-art sEV detection and diagnostic methods via surface protein markers will be introduced and discussed (Table 5).

**TABLE 5:** Summary of emerging sensing techniques for detection of sEV-associated surface biomarkers.

Emerging sensing techniques	Cell line sample type	Targeted surface markers	LOD	Cancer detected
Electrochemical sensors				
Dual magnetic nanoprobes and CRISPR-CHA in a electrochemical (cyclic voltammetry) assay (Yang et al., 2025)	BT-474 cell line	HER2 EpCAM	220 particles/mL	Breast cancer
High-resolution spiral microfluidic channel-integrated electrochemical (differential pulse voltammetry) device (Kwon et al., 2025)	Plasma	PD-1 PD-L1	1 × 10^4^ EVs/mL	Lung cancer
Electric field-resistant bubble-driven wash-free one-step EV assay via square-wave voltammetry detection (Zhang et al., 2025)	A549 cell line Plasma	EpCAM PD-L1	4.25 × 10^2^ EVs/mL 3.25 × 10^2^ EVs/mL	Lung cancer
Anion exchange membrane sensor (Maniya et al., 2024)	Blood	CD63 EGFR	30 EVs/μL	Glioblastoma
Fluorescence/Chemiluminescence-based Techniques				
Target recycling amplification based fluorescent aptasensor (Gu et al., 2025)	HGC-27 cell line Plasma	MUC-1 EpCAM PTK7 PD-L1	2.15 × 10^6^ EVs/μL	Gastric cancer
Chemiluminescence sandwich immunosensor (Wang M et al., 2024)	Serum	CD63 PD-L1	7.76 × 10^2^ EVs/μL	Lung cancer
Colorimetric assays				
Magnetic core-gold shell nanohybrids in a colorimetric sandwich magneto-immunoassay (Haizan et al., 2025)	MCF-7 cell line	CD24 CD9	37 particles/µL	Breast cancer
Multiple microarray analyzer (Jorgensen et al., 2025)	Plasma	TNF RII PP13 LFA1 ICAM CD81 CD82 Alix C1q	N/A	Preterm delivery Preeclampsia
MOF-aptamer-AuNPs-dopamine interaction-based platform for exosome capture and detection (Kuang et al., 2024)	Plasma	CD63	9.1 × 10^4^ particles/µL	Breast cancer
Centrifugal disk chip (Wang Y et al., 2024)	Blood	CD63 EpCAM PSMA HER2 EGFR CEA CA125	N/A	Breast cancer
Plasmonic sensors				
Sandwich-based SERS immunoassay of capture magnetic microbeads and detection SERS nanotags (Ngo et al., 2023)	Cell lines Plasma	EpCAM CA125 CD24	1.5 × 10^5^ particles/µL	Ovarian cancer
Brain nanoMET sensor (Premachandran et al., 2025)		PD-L1	N/A	Breast and lung cancer-metastasized brain tumors
Antibody-functionalized active whispering gallery mode microsphere microresonators (Suo et al., 2024)	Blood	MUC1 EGFR EpCAM	40 exosomes/probes	Breast cancer
Biological affinity-based sensors				
Quantifiable antibody-DNA conjugate-assisted quantitative methods combined with proximity ligation technology (Du et al., 2025)	HCT116 cell line A549 cell line	EpCAM	2.53 × 10^−4^ exosomes/µL (HCT116) 1.10 × 10^−4^ exosomes/µL	Lung cancer Cancerous tumors
Proximity ligation assay-induced rolling circle amplification for dual recognition (Xu et al., 2023)	MDA-MB-231 cell line HepG2 cell line	Glycosylated PD-L1 Glycosylated PTK7	1.04 × 10^4^ particles/mL (MDA-MB-231) 2.759 × 10^3^ particles/mL (HepG2)	Breast cancer Liver cancer

### 3.1 Electrochemical sensors

In sensing technology, electrochemical sensors stand out due to their simplicity, cost-effectiveness, and user-friendliness while still offering reasonable sensitivity and efficiency. Because of these advantages, electrochemistry is the most frequently applied mechanism for sEV detection. Nonetheless, it is important to keep in mind that the complex nature of clinical samples and high heterogeneity of EVs can be a challenge for electrochemical sensors in controlling non-specific bindings and background interferences.

**FIGURE 5:**
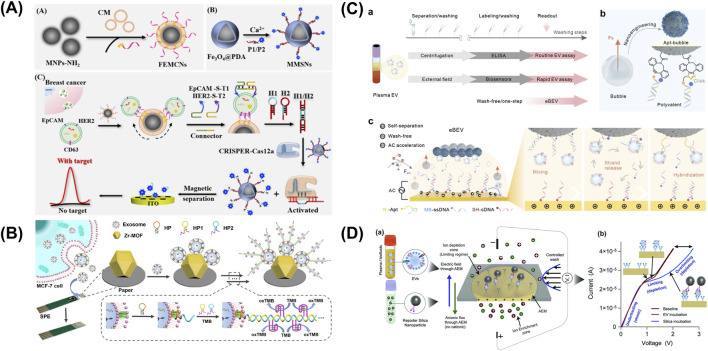
Electrochemical sensors targeting sEV-associated surface proteins. **(A)** A combination of dual magnetic nanoprobes and clustered regularly interspaced short palindromic repeats-catalytic hair pin assembly (CRISPR-CHA) in an electro-chemical assay for an ultrasensitive and high-precision detection of breast cancer cell-derived EVs (BC-EVs) via specific surface markers HER2 and EpCAM. **(B)** Differential Pulse Voltammetry (DPV)-based biosensor integrating metal-organic framework (MOF) functionalized paper and CD63-specific aptamer on a screen-printed electrode (SPE). **(C)** An electric field-resistant bubble-driven wash-free one-step EV assay. **(D)** An anion exchange membrane sensor for the detection of CD63/EGFR colocalization on EV surface, with capture antibody fixed to the sensor and reporter antibody conjugated on silica nanoparticles for a sandwich immunoassay. Figures reproduced with permission (Liu et al., 2021; Maniya et al., 2024; Yang et al., 2025; Zhang et al., 2025). Copyright 2021 American Chemical Society, 2025 American Chemical Society, 2024 Springer Nature.

A combination of dual magnetic nanoprobes and clustered regularly interspaced short palindromic repeats-catalytic hair pin assembly (CRISPR-CHA) in an electrochemical assay, developed by Yang et al., offered an ultrasensitive and high-precision detection of breast cancer cell-derived EVs (BC-EVs) via specific surface markers HER2 and EpCAM that reached a limit of detection (LOD) of only 220 particles/mL in validation with BT-474 EVs (Figure 5A) (Yang et al., 2025). First, CD63 targeting aptamer-functionalized fluidity-enhanced magnetic capture nanoprobes recognized and isolated BC-EVs for subsequent analysis. On the other hand, partially complementary P1/P2 duplex were adsorbed on the surface of the metal-mediated magnetic signal nanoprobes with P1 strand being tagged with methylene blue (MB). Following the binding of Apt-S-T probes with EV surface markers HER2 and EpCAM, CRISPR-CHA assay was initiated, producing programmable H1/H2 duplex needed for nonspecific cleavage of P1/P2 duplex. MB-labeled P1 ssDNA was released and accumulated on the electrode, generating amplified signals, whereas all magnetic nanoprobes were removed by magnetic separation.

An isolation detection platform named high-resolution spiral microfluidic channel-integrated electrochemical device (HiMEc) was proposed by Kwon et al. (2025). This approach consists of a sample treatment step by filtering out lipoprotein using antibody-functionalized microbeads, followed by sample injection, reconcentration, lipo-protein complex removal, and analyte sensing. This multi-step process was performed sequentially on a spiral microfluidic channel composed of two inlets, five helical loops, and two outlets. To optimize sEV separation performance, the channel dimensions were set to 600 μm × 50 µm (width × height), with a total channel length of 15.7 cm and the radius of curvature of 0.3–0.7 cm. On the sensing area, the lung cancer-associated EV capturing probes, anti-PD-1 and anti-PD-L1, were conjugated on the surface of two screen-print carbon sensors. After the addition of MB-CD63 aptamer, the detection of EVs was recognized via changes in Cyclic Voltammetry measurements and corresponded to a concentration range of 1 × 10^4^ to 1 × 10^8^ EVs/mL.

Liu et al. reported a Differential Pulse Voltammetry (DPV)-based biosensor integrating metal-organic framework (MOF) functionalized paper and CD63-specific aptamer on a screen-printed electrode (SPE) (Figure 5B) (Liu et al., 2021). In this work, Zr-MOFs enable the sensitive capture of phosphate rich EV via Zr-O-P bonds, while aptamer amplifies via hybridization chain reaction upon binding, leading to the production of TMB-reducing DNAzyme. As higher concentration of free TMB induces higher DPV signal, successful sEV capture would lead to a decrease in free TMB, thus generating a lower DPV current. This enzyme-free, label-free paper-based system achieved a low LOD of 5 × 10^3^ particles/mL.

Using Square-wave Voltammetry (SWV) detection approach, Zhang et al. proposed an electric field-resistant bubble-driven wash-free one-step EV assay as a sEVs profiling platform (Figure 5C) (Zhang et al., 2025). In this work, an aptamer duplex was formed from an azide (N3)-modified EV-responsive DNA aptamer and a redox MB-labeled single-stranded DNA (MB-ssDNA) to subsequently assemble on synthesized click bubbles. Under alternating current, electroactive sequence MB-ssDNA separates from the aptamer duplex to hybridize with a capture probe (SH-cDNA) in the presence of sEVs, thus providing a SWV electrochemical signal readout. Plasma samples from healthy donors and lung cancer patients were successfully identified with high accuracy (>95%).

Maniya et al. presented an anion exchange membrane (AEM) sensor for the detection of CD63/EGFR colocalization on EV surface, with capture antibody fixed to the sensor and reporter antibody conjugated on silica nanoparticles for a sandwich immunoassay (Figure 5D) (Maniya et al., 2024). In an ion-exchange membrane, the over limiting current transition voltage caused by ion-depletion action at its highest point is sensitive to the membrane charges. Taking advantage of this regime, charges influenced by capture antibody-EV-silica nanoparticle immunocomplexes can be accurately quantified through distinctive voltage shift signals. A specialized biochip, with the dimensions of 25 mm width and 54 mm length, was fabricated to house the AEM membrane, inlet and outlet ports and a microfluidic channel (3 mm × 35 mm × 250 μm, width × length × height). The platform reported a detection range of as low as 30 to 300,000 EVs/mL and was validated using clinical samples of GBM.

### 3.2 Fluorescence/chemiluminescence-based techniques

Optical sensing techniques rely on the changes upon interaction of light when projecting onto analytes. Among these sensors, fluorescence and chemiluminescence-based sensors provide highly sensitive detection via direct visualization. Gu et al. introduced a rapid target recycling amplification based fluorescent aptasensor for cancer EVs detection with assay time less than 40 min (Gu et al., 2025). The hairpin probe, labeled with a fluorophore at 5′ terminal and a quencher at 3′ terminal, maintained its double-stranded structure without the availability of cancer-derived EVs, hence produced no signals; however, upon binding with EVs, the probe unwound and released fluorescence. The complementary enzyme exonuclease I could now digest the opened ssDNA into nucleotides, leading to a replacement of new hairpin probes and recycling of signal amplification. The method reported a LOD of 2.15 × 10^6^ particles/µL using MUC1 probe for gastric cancer cell line HGC-27-derived EV sample, as well as validation for other gastric cancer biomarkers (EpCAM, PTK7, PD-L1) for both cell line EV isolates and human plasma specimens.

**FIGURE 6:**
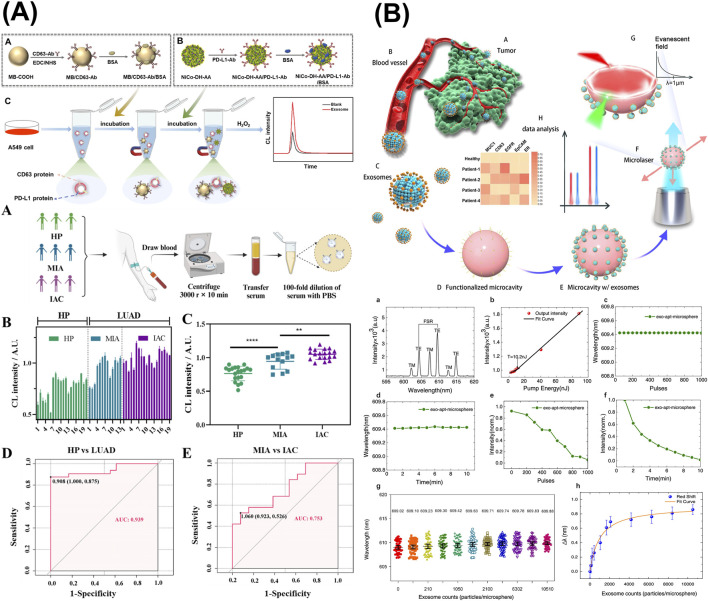
Optical sensors targeting sEV-associated surface proteins. **(A)** A chemiluminescence (CL) sandwich immunosensor consisting of N-(4-aminobutyl)-N-ethylisopropanol-functionalized nickel–cobalt hydroxide (Ni-Co-DH-AA) nanoflower probe conjugated with PD-L1 and anti-CD63-AuNPs for diagnosis of lung adenocarcinoma (LUAD). **(B)** An antibody-functionalized active WGM microsphere microresonators as breast cancer-derived sEV detection system via MUC1, EGFR, and EpCAM. Figures reproduced with permission (Suo et al., 2024; Wang M. et al., 2024; Yang et al., 2025). Copyright 2024 American Chemical Society.

Wang et al. developed a chemiluminescence (CL) sandwich immunosensor consisting of N-(4-aminobutyl)-N-ethylisopropanol (ABEI)-functionalized nickel–cobalt hydroxide (Ni-Co-DH-AA) nanoflower probe conjugated with PD-L1 and anti-CD63-AuNPs for diagnosis of lung adenocarcinoma (LUAD) (Figure 6A) (Wang M. et al., 2024). NiCo-DH-AA were synthesized starting from NiCo-glycerate sphere, which was then subjected to hydrolysis, AuNP decoration, and ABEI modification. The final nanostructure NiCo-DH-AA exhibits excellent CL emission due to the excited state of ABEI oxidized products, serving as the signal probe in this immunoassay. This approach delivered a detection range of 4.75 × 10^3^–4.75 × 10^8^ particles/µL, with an LOD of 7.76 × 10^2^ particles/µL, successfully identified LUAD patients and correctly classified stagings from serum samples.

### 3.3 Colorimetric assays

Similarly, colorimetric optical sensors also allow naked-eye readout with excellent sensitivity. A study aiming for breast cancer diagnosis employed nanomagnetic core-gold shell nanohybrids (mAuNHs) in a colorimetric sandwich magneto-immunoassay (mLISA) (Haizan et al., 2025). Anti-CD24 antibodies were immobilized in the microtiter plate to capture breast cancer cell line MCF7-derived EVs, followed by the introduction of anti-CD9 mAuNHs as detection probe into the assay. After magnetic separation to remove unbound mAuNHs, and colorimetric readout was established using TMB substrate. This mLISA platform achieved an LOD as low as 37 particles/µL and successfully distinguished MCF7-derived exosomes from other cell lines-derived ones.

Jorgensen et al. evaluated a Multiple Microarray analyzer for prediction of preterm delivery (PTD) and PE via total and placental-derived EVs (Jorgensen et al., 2025). After printing the antibodies on an epoxy-coated slide, the detection was carried out in a similar manner to a standard sandwich ELISA. Data was analyzed in pairs to identify the best performing combinations for PTD and PE diagnosis.

An extended study from the work of Liu et al. (2021) introduced an sEV sensing system using AuNPs as colorimetric indicator based on dispersed or aggregated state (Kuang et al., 2024). In this design, sodium tripolyphosphate was added to block unbound areas on Zr-MOF, preventing DNA aptamer absorption on Zr-MOF, thereby increasing the affinity to AuNPs. In the presence of dopamine, the more EVs captured, the more DNA aptamer binds to AuNPs, thus the higher the resistance of AuNPs to aggregation. This approach achieved a detection limit of 9.1 × 10^4^ particles/µL.

Wang et al. developed a rapid, automated, smartphone-integrated EV separation and detection on a centrifugal disk chip with whole blood sample (Wang Y. et al., 2024). The PDMS-on-glass platform consisted of two pairs of identical units, each containing sets of inlets and outlets and automatic venting channel at the end. Within the unit, each structural part corresponded to a step in the process: decanting structure (big blood cell removal), siphon valve (collecting preprocessed liquid), triangular array region (size-exclusion blood cell removal, pure plasma collection), and detection chamber (EV sensing). In detail, the triangular column array region achieved successfully cell removal by gradient gaps of 0.2 mm, 0.1 mm, and 0.05 mm, and the threshold capillary force used to drive the fluid flow into the siphon was calculated to optimize centrifugal speeds applied in each process. Based on the sandwich ELISA principle, isolated EVs were captured by anti-CD63 modified magnetic beads and subsequently bound by breast cancer-associated markers (EpCAM, PSMA, HER2, EGFR, CEA, CA125) serving as detection probes, with sensing completed via TMB oxidation-induced color change. The results suggest that the combined analysis of CEA, CA125, and EGFR demonstrated superior performance in cancer progression monitoring than individually assessed markers, with expression levels increasing over time.

### 3.4 Plasmonic sensors

#### 3.4.1 Raman spectroscopy sensors

Raman spectroscopy can be considered as one of the major breakthroughs in biomedical sensing advancement (Ferreira et al., 2024). Many of its variants contribute tremendously to the development of highly accurate sensors for clinical applications, with the most notable being SERS. SERS is especially suitable for sEVs detection by offering excellent sensitivity, high throughput, and multiplexing potential; however, this technology heavily depends on the quality of plasmonic materials. Therefore, either improvement of SERS substrates in label-free SERS or implementation of SERS probes in label-based SERS is the ultimate key to overcome this challenge (Chen and Qiu, 2025).

Ngo et al. optimized a sandwich-based SERS immunoassay employing tetraspanin-functionalized capture magnetic microbeads and EpCAM/CA125/CD24-modified SERS nanotags for the design of an ovarian cancer diagnostic test, which eventually achieved a minimum detection concentration of 1.5 × 10^5^ particles/µL (Ngo et al., 2023).

Premachandran et al. established the Brain nanoMET sensor for the detection of breast and lung cancers-metastasized brain tumors EVs (Premachandran et al., 2025). The sensor, which was fabricated by an ultrashort femtosecond laser ablation process, provided highly effective SERS for characterization of EV molecular profiles. Validation with the surface marker PD-L1 resulted in expression variations between primary tumor-derived and metastatic EVs, proving the potential of this method as a diagnostic tool.

#### 3.4.2 Whispering gallery mode sensors

Whispering gallery mode (WGM) resonators are a type of micro-engineered sensors that provide sensitive, label-free sensing of biochemical analytes in a timely manner. These optical microcavities trap and let electromagnetic waves oscillate within their inner structures, which in turn alters the electromagnetic field and subsequently leads to the formation of an evanescent field. In the presence of target molecules, the binding will induce a change in effective refractive index, a parameter that is measurable via resonance wavelength shift. Despite the impressive sensitivity, this method is prone to background noise, i.e., thermo-optic and thermo-mechanic effects (Righini et al., 2011). To address this drawback, Suebka et al. described a new method, frequency locked whispering evanescent resonator, which utilized the ultra-sensitive microtoroid variant of WGM sensors. In this technique, frequency locking served as a noise reduction add-on, enabling the potential detection of single protein molecules or exosomes (Su et al., 2016; Suebka et al., 2025).

Utilizing antibody-functionalized active WGM microsphere microresonators, Suo et al. proposed a breast cancer-derived sEV detection system that achieved an LOD of 40 exosomes per probe (Figure 6B) (Suo et al., 2024). In the experimental setup, the WGM microresonator was laser-illuminated, triggering microlaser emission, which in turn created an evanescent field surrounding the core microsphere. The validated breast cancer surface marker panel consisted of MUC1, EGFR, and EpCAM. Subsequently, the binding of exosomes via modified antibodies on the microresonator’s surface would cause a wavelength shift, hence exosome concentration can be derived.

### 3.5 Biological affinity-based sensors

Antibody or aptamer-antigen affinity is vital for immunosorbent detection of sEV-associated surface proteins. Despite the excellent sensitivity and strength of this biological binding, the relatively low abundance of sEV membrane proteins requires effective signal amplifying strategies. To counter this setback, several studies have introduced DNA sequences as signaling probes and attempted various DNA replication techniques, ranging from the most basic PCR to more advanced RCA.

**FIGURE 7:**
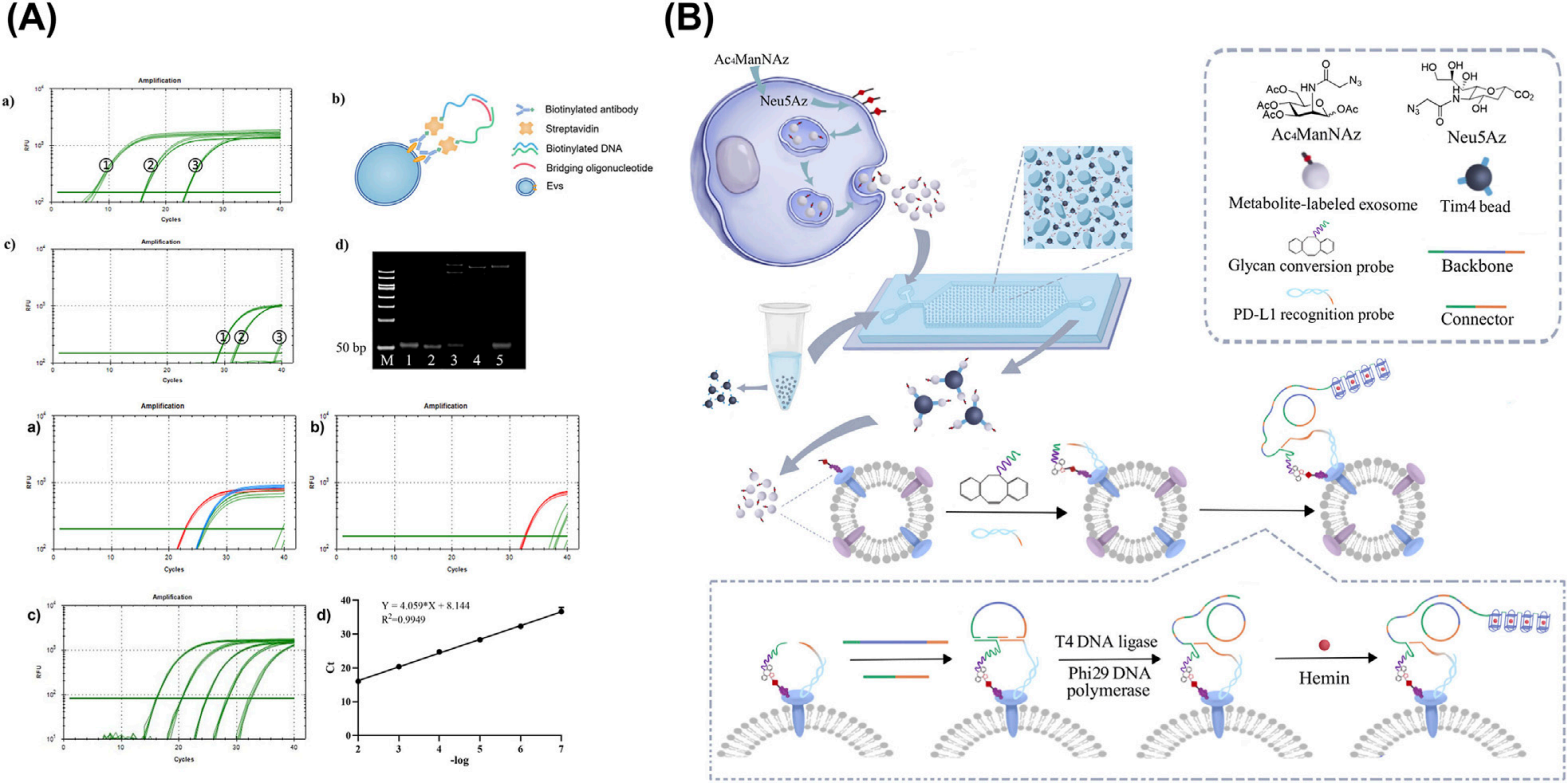
Biological affinity-based sensors targeting sEV-associated surface proteins. **(A)** An assay combining the TAEs capture via biotinylated anti-EpCAM subunit with the signal amplification of oligonucleotides through qPCR using the PL method. **(B)** Detection of glycosylated protein markers via dual-aptamer assembly for PL and RCA. Figures reproduced with permission (Xu et al., 2023; Du et al., 2025). Copyright 2023 American Chemical Society, 2025 MDPI.

An approach combining the power of affinity antibodies and amplifiable oligonucleotides was developed by Du et al., in which antibody-DNA conjugate was constructed to quantify surface proteins in tumor-associated EVs (TAEs) (Figure 7A) (Du et al., 2025). After the capture of TAEs via biotinylated anti-EpCAM subunit, the three attached DNA sequences were amplified through qPCR using the proximity ligation (PL) method. As a result, with a DNA concentration of 100 pM, the concentrations of exosomes derived from EpCAM-positive cell lines HCT116 and A549 were reported at 2.53 × 10^−4^ and 1.10 × 10^−6^ exosomes/mL, respectively.

A similar study based on PL assay was completed by Xu et al., with a design to combine selective aptamers with RCA, highlighting its advantages of being isothermal, highly sensitive, specific, and reproducible compared to other DNA replication methods (Figure 7B) (Xu et al., 2023). Aside from the surface protein-capture aptamer, glycan conversion probe for glycosylated molecule recognition was also included to create a dual-probe assembly for PL, thereby triggering RCA. As a result, tandem G-quadruplex sequence was amplified and detectable via absorbance at 460 nm upon incubation with hemin, ABTS, and H_2_O_2_. The isolation of exosome subpopulations was performed on a microfluidic chip, consisting of a 70-µm high drop-shaped micropillar arrays. Samples and reagents were loaded via programmable microinjection pump using optimized flow rate of 0.2 μL/min (Zheng et al., 2022). Through this setup, highly glycosylated surface protein markers were detected, achieving LODs of 1.04 × 10^4^ and 2.759 × 10^−4^ particles/mL for PD-L1 on MDA-MB-231 cell line-derived EVs and PTK7 on HepG2 cell line-derived EVs, respectively.

## 4 Challenges and future perspectives

Cancer has always been the leading cause of death worldwide. One of the major contributing causes is the lack of a rapid, robust, reliable, and affordable diagnostic measure. To this day, the gold standard for confirming cancer remains tissue biopsy, a highly invasive and time-consuming procedure which requires skilled personnel for operation, making it less accessible to people in need. The alternative method, liquid biopsy, reduces invasiveness by analyzing circulating tumor cells (CTCs) and molecules in bodily fluids. Among these analytes, sEVs and exosomes have emerged as the newest targets for diagnosis of cancer, especially during the early stages of the disease.

The role of sEVs and exosomes in cancer progression and tumorigenesis has been rigorously investigated in recent years. These special vesicles are known to bear molecular characteristics from their own parental cells, serving as indicators of illnesses. Development of sensitive sEV sensing devices or assays is a promising approach towards more efficient, timely diagnosis of cancer. However, this is a complex endeavor that spans several key aspects: (1) the alignment and standardization of sEV methodologies, (2) the biological and technical challenges of sEV-associated surface proteins as detection targets, (3) the expansion of diagnostic scope and biosensing ability of sEV-associated biomarkers, and (4) the establishment of global regulatory frameworks.

### 4.1 Standardization of sEV methods and quality control

A significant challenge stemming from the very beginning of the sEV handling is selecting the appropriate method of isolation. Currently, there is no global consensus on a single method for sEV enrichment among researchers around the world. Similarly, various techniques, both existing conventional methods and newly developed tools, are employed for sEV characterization without a global common ground. These differences might contribute to the varied and contrasted findings in the literature (Baranyai et al., 2015; Takov et al., 2019; Mukerjee et al., 2025) It is crucial to implement a standardized framework on sEV processing, ensuring biosensing performance across essential parameters such as sensitivity, accuracy, reproducibility, and clinical reliability.

On the other hand, sEV-encapsulated molecules are undeniably a valuable source of information regarding the state of diseases and cancer. Numerous studies on the role of these sEV internal cargos in pathological pathways have revealed a multitude of molecules as disease-specific biomarkers. A compulsory step in these studies prior to downstream analysis is sEV lysis, in which specialized protease agents disrupt the sEV membrane, allowing the release of exosomal proteins and nucleic acids from original vesicles. This addition to the workload is not recommended for real-world clinical scenarios where portable, minimally processed POC approaches are preferred. Moreover, it is strictly important to monitor the purity of sEV isolates before lysis. High concentrations of non-sEV impurities or biological contamination would significantly affect the final yield post-lysis process, thereby hampering the downstream analysis results.

### 4.2 Overcome limitations of sEV-associated surface protein markers in diagnostic device development

To bypass sEV lysis, direct investigation of exosomal surface molecules is a reasonable alternative. The membrane of sEV strongly resembles the cell membrane, consisting mainly of a lipid bilayer and embedded proteins. Considering molecular specificity, sEV surface proteins emerge as a potential target for sensor design and development.

However, there are persisting technical challenges in detection of sEV-associated protein biomarkers. First, protein structures are mostly buried in the lipid bilayer with limited epitope exposure to the sensing environment. Furthermore, due to the heterogeneous nature of sEVs, the expressions of the associated biomarkers vary from sample to sample, vesicle to vesicle, which might lead to inconsistent reports among research groups and raise a concern regarding batch variability. Lastly, they have relatively lower abundance, especially in earlier disease stages, causing difficulty in developing an effective disease screening platform. These problems lead to significant setbacks in binding with capturing and detection probes, requiring strategies to enhance the efficiency of these interactions. In this paper, sEV surface protein-targeted profiling methods and diagnosis-aiding sensing platforms have been introduced and discussed. While molecule-specific procedures, such as ELISA and WB, are transferable to sEV studies, the major characteristic differences between free molecular entities and intact vesicles create a significant gap in their direct application to sEV experiments. To overcome this limitation, various sensor designs, ranging from electrochemical, optical, to biological affinity-based principles, have been developed in past years and achieved successful detection of surface proteins from sEVs, emphasizing the potential of this particular class of disease markers.

There are several trends in sEV-associated surface protein profiling and sensing platform development. For instance, emerging single-vesicle sensing strategies allow for higher sensitivity and analysis at low concentration while requiring minimal volume of initial sample (Amrhein et al., 2023). Moreover, multiplexing capacity (Suo et al., 2024), high-throughput setup (Wu et al., 2019), and assessing combinations of protein markers (Weber et al., 2023b; Martorana et al., 2024) also enable enhanced accuracy, rapid handling, and highly efficient methods, which are desirable properties of POC devices. Finally, the massive growth and cross-field application of machine learning and artificial intelligence (AI) also contribute to sEV surface protein profiling and sensing technology, opening the door to predictive and automated diagnostic systems (Gu et al., 2025; Premachandran et al., 2025; Zhang et al., 2025). In surface protein identification, with the power of AI models, meaningful features can be extracted for a reliable prediction of diagnosis-relevant sEV surface biomarkers from general EV proteomic profiles, thereby facilitating the biomarker discovery process. Moreover, direct integration of AI functions in diagnostic tools is highly desirable due to the flexibility in pattern analysis towards different types of data, from region of interest in image analysis to molecular concentrations in biochemical assays. Together, these cutting-edge approaches have enhanced the detection efficacy and reliability, forming a solid foundation for future clinical adaptation.

### 4.3 Expansion of diagnostic potential of sEV-associated surface biomarkers

The future direction of sEV-associated surface biomarker-based detection should prioritize expanding the range of target diseases as well as diversifying the types of sampling bodily fluids, as current research remains heavily focused on cancer diagnostics via blood analysis. Although blood has been the primary and most established source for liquid biopsy as it contains most of the standard analytes for cancer diagnosis, especially CTCs, the process of blood drawing is invasive, requires trained personnel, and can be uncomfortable for patients. Developing diagnostic tools to assess different non-invasive bodily fluids, such as urine, saliva, etc., would greatly encourage self-operated and contactless sample collection.

On the other hand, due to the large size of CTCs, their concentration in these fluids is significantly lower, while cells found in urine or saliva typically characterize only localized lesions. Shifting towards non-blood fluids would advance the growth of nanoscale molecular sensing, including sEV-based diagnostics due to their ability to cross stringent physiological barriers, such as blood-brain and blood-retinal barriers. It is crucial to keep note that the exterior of sEV membrane does not house only proteins. In a broader sense, sEV surfaceome is not only about surface proteins but also specific molecular modifications on the sEV membrane, such as glycans. These extracellular moieties are actively being investigated in recent years as promising disease hallmarks, expanding the current knowledge on sEV surfaceome and presenting opportunities to develop highly sensitive sensors (Shimoda et al., 2019; Xu et al., 2023).

Consequently, sEVs and their membrane-embedded biomarkers may offer tremendous diagnostic potential beyond current achievements. To fully realize this, further sEV biological research is required to identify the relationship, related mechanisms and pathways linking these secreted vesicles and their membrane-bound biomarkers to various pathological conditions.

### 4.4 The need for global regulations for translation into clinical practice

Beyond the technical limitations, the translation of biosensing technologies via sEV-associated surface biomarkers from bench to bedside also faces difficulties related to global regulatory alignment, similar to those encountered in the clinical adoption of EV-based therapeutics. A review paper addressing these challenges and offering counter strategies, although focused on EV therapeutic products, discussed many of the same crucial frameworks such as protocol standardization, quality assurance, and efficacy control, that are applicable to the advancement of diagnostic devices (Verma and Arora, 2025). Nevertheless, it remains essential to develop dedicated guidelines for regulatory harmonization regarding sEV-related sensing advancements and their clinical translation.

The potential of sEVs in clinical practice, including both diagnostics and therapeutics, has driven rapid innovation in this field in recent years, contrasting sharply with the comparatively slow establishment of global regulatory policies. This mismatch raises a broader dilemma: when faced with clinical urgency, how can life-saving diagnostic tests reach the patients in a timely manner, without breaching the regulations? An unprecedent real-world example is the temporary implementation of the Emergency Use Authorizations to facilitate the approval of new clinical tools, which was an effort to counter the COVID-19 pandemic. Therefore, only when both technical and regulatory challenges are properly addressed can these technologies be successfully translated into clinical practice, ensuring the benefits for patients in need.

## 5 Conclusion

Liquid biopsy of circulating sEVs is a promising clinical technique for sensitive and early diagnosis of diseases, including cancers. This review discusses the diagnostic value of sEVs and their associated molecules, especially the lesser-known sEV membrane embedded proteins. Beyond total sEV quantification and internal cargo profiling, assessment of sEV-associated surface proteins provides a lysis-free and robust approach while still maintaining the diagnostic relevance. In addition, we reviewed state-of-the-art sEV-specific proteomic analytic approaches and sEV surface biomarker-targeted sensors were introduced, emphasizing the feasibility and advancement of these strategies towards the diagnostic goal.

Nonetheless, major challenges stemming from limited exposure of specific epitopes and relatively low abundance nature of the target require continuous efforts. The future outlook is positive with several development approaches involving both fundamental and technical innovations, such as expanding disease surfaceomic molecule panels, thorough validation studies, incorporating multiplexed detection, enabling high-throughput in microfluidic platforms, and AI-aided analytical pipelines. With these combined efforts, the diagnostic technology based on sEV-associated surface biomarkers is very near its clinical realization, shifting its significance from lab benches to patients’ bedside.
