# Supplementary material for: Small extracellular vesicle-associated surface protein biomarkers: emerging roles, opportunities, and challenges in diagnostics
Source: Front Bioeng Biotechnol. 2025 Dec 1;13:1714972. doi: 10.3389/fbioe.2025.1714972 (PMC12702871; doi:10.3389/fbioe.2025.1714972)
Supplement: Supplementary file 1 [file Supplementaryfile1.docx]

Small extracellular vesicle-associated surface protein biomarkers: emerging roles, opportunities, and challenges in diagnostics

Thi Thanh Huong Pham^1^, Hiroaki Sakamoto^2^, Tatsuhito Hasegawa^3^, Chisato Sakamoto^4^, Shin-ichiro Suye^5^, Han-Sheng Chuang^1,6*^

^1^Department of Biomedical Engineering, National Cheng Kung University, Tainan, Taiwan

**^2^**Department of Frontier Fiber Technology and Science, Graduate School of Engineering, University of Fukui, Fukui, Japan

**^3^**Fundamental Engineering for Knowledge-Based Society, Graduate School of Engineering, University of Fukui, Fukui, Japan

**^4^**Department of Chemistry and Biology, National Institute of Technology, Fukui College, Fukui, Japan

**^5^**Division of Engineering, Faculty of Engineering, University of Fukui, Fukui, Japan

^6^Medical Device Innovation Center, National Cheng Kung University, Tainan, Taiwan

***Correspondence:**Han-Sheng Chuang
oswaldchuang@mail.ncku.edu.tw

Supplementary Material

**
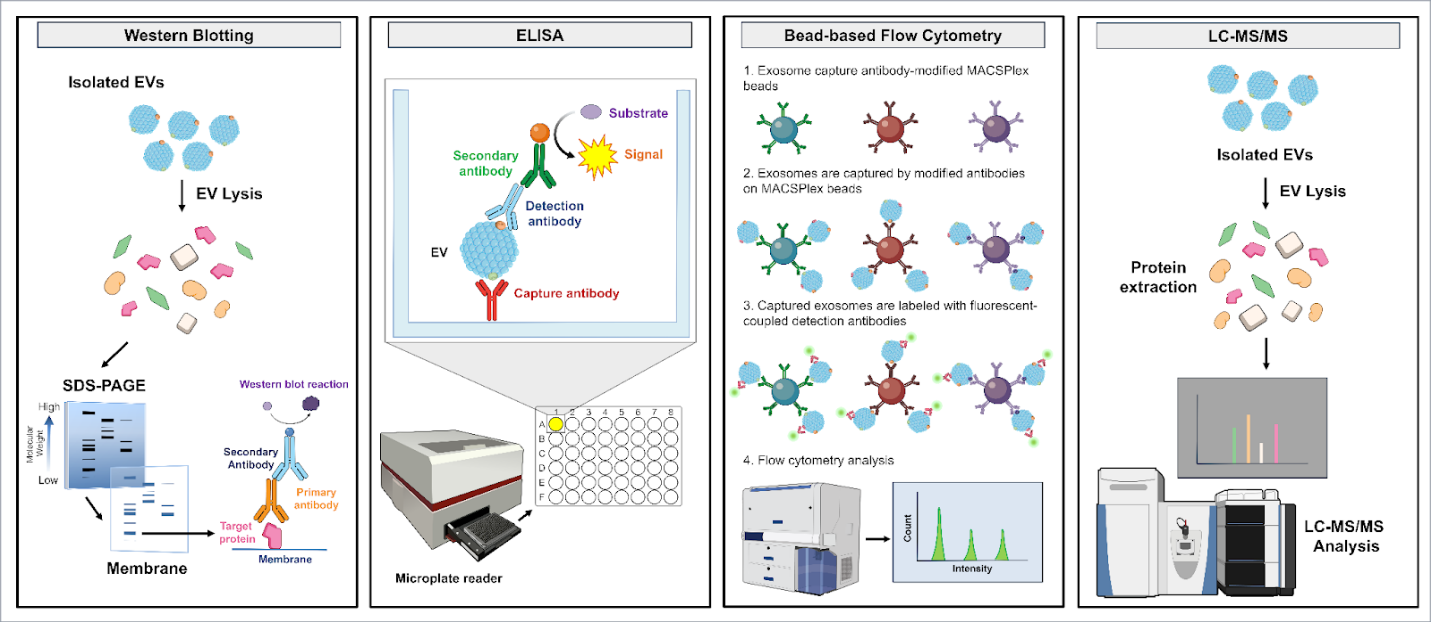
**

**Supplementary Figure 1.** Conventional sEV-associated surface protein profiling. Bulk immunoassays, such as WB and ELISA, are straightforward and well-established as a protein identification and quantification tool. On the other hand, bead-based flow cytometry is an effective method in which a multiplexed approach is enabled. For a label-free and high-throughput approach, LC-MS/MS provides a thorough protein profile of the EV samples. Art visual elements reproduced from NIAID NIH BIOART Source. Public domain.
